# Androgen Signaling Disruption during Fetal and Postnatal Development Affects Androgen Receptor and Connexin 43 Expression and Distribution in Adult Boar Prostate

**DOI:** 10.1155/2013/407678

**Published:** 2013-09-17

**Authors:** Anna Hejmej, Ewelina Górowska, Małgorzata Kotula-Balak, Katarzyna Chojnacka, Marta Zarzycka, Justyna Zając, Barbara Bilińska

**Affiliations:** Department of Endocrinology, Institute of Zoology, Jagiellonian University, Gronostajowa 9, 30-387 Krakow, Poland

## Abstract

To date, limited knowledge exists regarding the role of the androgen signaling during specific periods of development in the regulation of androgen receptor (AR) and connexin 43 (Cx43) in adult prostate. Therefore, in this study we examined mRNA and protein expression, and tissue distribution of AR and Cx43 in adult boar prostates following fetal (GD20), neonatal (PD2), and prepubertal (PD90) exposure to an antiandrogen flutamide (50 mg/kg bw). In GD20 and PD2 males we found the reduction of the luminal compartment, inflammatory changes, decreased AR and increased Cx43 expression, and altered localization of both proteins. Moreover, enhanced apoptosis and reduced proliferation were detected in the prostates of these animals. In PD90 males the alterations were less evident, except that Cx43 expression was markedly upregulated. The results presented herein indicate that in boar androgen action during early fetal and neonatal periods plays a key role in the maintenance of normal phenotype and functions of prostatic cells at adulthood. Furthermore, we demonstrated that modulation of Cx43 expression in the prostate could serve as a sensitive marker of hormonal disruption during different developmental stages.

## 1. Introduction

The prostate, an accessory gland of the male urogenital system, is under strict control by testicular hormones and androgens. Androgens, apart from maintaining tissue homeostasis and controlling secretory function of prostatic epithelium, play a major role in the regulation of prostate development and differentiation before birth and during puberty. The majority of the effects of androgens are mediated by the androgen receptor (AR), a member of ligand-activated nuclear receptors family [1]. Inactivating mutations of the AR or exposure to high doses of AR antagonists during in utero development have been shown to severely compromise prostate growth and differentiation, resulting in its complete agenesis or marked decrease in prostate weight at birth [2–4]. On the other hand, transient disruption of androgen action during some specific periods of fetal or postnatal development induces functional alterations that may not be manifested until puberty or afterwards [5–7]. For example, gestational exposure of rats to an antiandrogen, vinclozolin, during gonadal sex determination results in adult onset disease of the prostate. Prepubertal rats showed no abnormalities in ventral prostate morphology, whereas in the older animals regression of prostatic secretory epithelium, cystic hyperplasia, and focal prostatitis were detected [5, 6].

Recent studies from this laboratory showed that transient fetal, neonatal, or prepubertal exposure to an antiandrogen flutamide leads to adverse effects on the morphology and hormonal functions of the testis and epididymis of adult boar. Furthermore, these alterations were associated with changes in the expression and distribution of intercellular junction proteins, including gap junction protein connexin 43 (Cx43) [8–10].

Cx43 belongs to the multigene connexin family that consists of at least 21 members in humans [11]. In the plasma membrane of two neighboring cells connexin protein subunits form hemichannels called connexons. Gap junction channels are composed of two identical (homotypic) or different (heterotypic) connexons and allow a direct exchange of small molecules (<1 kDa) such as nucleotides, second messengers, peptides, and ions between the cells [12]. Recent findings have demonstrated that gap junctions composed of Cx43 have an essential role in the regulation of cell adhesion, proliferation, differentiation, homeostasis, and oncogenic transformation in male reproductive system [13].

Studies in rodents showed that in the prostate different types of cells communicate via gap junctions composed of different connexins. During early steps of postnatal development Cx43 expression is strong in periurethral mesenchymal cells and undifferentiated epithelial cells. As the process of morphogenesis continues, Cx43 expression declines, whereas Cx32 expression increases concomitantly with an increase in the differentiated secretory cell population. Finally, in the rat adult prostate the expression of Cx32 is restricted to secretory epithelial cells, whereas basal epithelial cells express Cx43 [14, 15]. Dysregulation of these Cxs expression, especially of Cx43, is thought to play a role in carcinogenesis [16, 17]. 

Although it was reported that the Cx43 expression, trafficking, and assembly in the prostatic cells are influenced by the androgens, limited knowledge exists regarding role of the androgen signaling during specific periods of prenatal and postnatal development on their expression in adult prostate. Therefore, in this study we examined Cx43 expression and distribution in adult pig prostate following fetal, neonatal, and prepubertal exposure to flutamide. Moreover, to answer the question whether androgen signal was altered in the adult boar prostates, the expression of androgen receptor was evaluated. In addition, the effect of antiandrogen on prostatic tissue morphology, apoptosis, and proliferation of prostatic cells was analyzed.

## 2. Material and Methods

### 2.1. Animals and Experimental Design

Sexually mature boars (Large White × Polish Landrace) originating from five litters were allotted into four groups (*n* = 3 each group). First group of experimental animals was exposed from day 20 to 28 of gestation (GD20) to an antiandrogen flutamide (2-methyl-N-[4-nitro-3-(trifluoromethyl)-phenyl]propamide; Sigma-Aldrich, St Louis, MO, USA). Next two groups were injected with flutamide postnatally on days 2–10 (PD2) or on days 90–98 (PD90) after birth. Flutamide was suspended in corn oil and administered subcutaneously in five doses (50 mg/kg body weight) every second day to antagonize testosterone action without producing a toxic effect in the sow and neonates [3]. The last group included control animals for each experimental group. Control boars were given a vehicle only (corn oil). The time of exposure to flutamide was based on the literature and our own data described previously [10, 18, 19]. Briefly, in utero exposure to flutamide included the period of embryonic gonadal sex determination, whereas postnatal exposures included the periods of neonatal and prepubertal development [20, 21]. Both control and experimental animals were maintained under identical conditions, with *ad libitum* feeding and water until 9 months of age when they were slaughtered and prostates were removed. All surgical procedures were performed by a veterinarian and followed approved guidelines for the ethical treatment of animals in accordance with the Polish legal requirements under the license given by the Local Ethics Committee at the Jagiellonian University in Krakow (no. 34/2008).

### 2.2. Tissue Preparation

Tissue samples were fixed by immersion in 4% formaldehyde freshly prepared from paraformaldehyde and dehydrated in an increasing gradient of ethanol, cleared in xylene, and embedded in paraplast (Sigma-Aldrich) for immunohistochemistry. Sections of 5 *μ*m in thickness were mounted on slides coated with 3-aminopropyltriethoxysilane (Sigma-Aldrich), deparaffinized, and rehydrated through decreasing alcoholic solutions. Other tissue fragments were immediately frozen in a liquid nitrogen and stored at −80°C for RNA isolation and protein extraction.

### 2.3. RNA Isolation, Reverse Transcription, and RT-PCR

Total RNA was extracted from prostates using TRIzol reagent (Life Technologies, Gaithersburg, MD, USA) according to the manufacturer's instruction. To remove residual DNA contamination, the RNA samples were incubated with RNAase-free DNAse (Promega, Madison, WI, USA) at 37°C for 15 minutes. The yield and quality of the RNA was assessed by measuring the *A*_260_ : *A*_280_ ratio in a spectrophotometer (NanoDrop 2000, Thermo Scientific, Wilmington, DF, USA) and by electrophoresis. 

The purified total RNA was used to generate cDNA. A volume equivalent to 1 *μ*g of total RNA was reverse transcribed using High-Capacity cDNA Reverse Transcription Kit (Applied Biosystems, Carlsbad, CA, USA) according to the manufacturer's protocol. cDNA was prepared in a 20 *μ*L volume using the random primers, dNTP mix, RNAse inhibitor, and reverse transcriptase (RT). Parallel reactions for each RNA sample were run in the absence of RT to assess any genomic DNA contamination. 1 *μ*L of RNase-free water was added in place of RT. 

The cDNA samples were then subjected to PCR amplification performed in a Veriti thermal cycler (Applied Biosystems) with a temperature cycling program of 10 min at 25°C, 2 h at 37°C, and 5 min at 85°C. Samples were kept at −20°C until further analysis. PCR reactions were performed with reaction mixture containing 1 *μ*L of cDNA, 10 *μ*M forward and reverse primers obtained from Institute of Biochemistry and Biophysics PAS (Warsaw, Poland), 10 mM of dinucleotide triphosphate, 10x PCR buffer, and 2 units of DyNAzyme II polymerase (Finnzymes, Espoo, Finland) in a Veriti thermal cycler. The primer sequences used for PCR amplifications were as follows: for AR, forward (5′-CACATTGAAGGCTATGAGTG-3′) and reverse (5′-CCCATCCAGGAGTACTGAAT-3′) [22]; for Cx43, forward (5′-GGT GGA CTG TTT CCT CTC TCG-3′) and reverse (5′-GGA GCA GCC ATT GAA ATA AGC-3′); and for glyceraldehyde-3-phosphate dehydrogenase (GADPH) forward (5′-GGA CTC ATG ACC ACG GTC CAT-3′) and reverse (5′-TCA GAT CCA CAA CCG ACA CGT-3′) [23]. GAPDH was used as an internal control. No-RT controls were performed for each RNA sample and no-template controls were included on each PCR plate.

Three independent experiments were performed. All PCR products were analyzed by electrophoresis on 1.5–2.5% agarose gels with ethidium bromide together with a ready-load 100 bp DNA ladder marker (Promega) and followed by fluorescence digitization using a Bio-Rad Gel Doc XR system (Bio-Rad Labs., Hercules, CA, USA).

### 2.4. Real-Time Quantitative RT-PCR

Real-time RT-PCR analyses were performed using the StepOne Real-Time PCR system (Applied Biosystems) with the same cDNA templates as described above. The mRNA expression levels of the AR and Cx43 were quantified in each sample using TaqMan Gene Expression Assays (Applied Biosystems) as follows: for AR assay ID, Ss03822350_s1; for Cx43 assay ID, Ss03374839_u1. GAPDH levels were determined as an endogenous control assay (Applied Biosystems, assay ID, Ss03375629_u1). Quantitative PCR was performed with 200 ng of cDNA, 1 *μ*L TaqMan PCR master mix (Applied Biosystems) in a final volume of 20 *μ*L. After 2 min of incubation at 50°C, the thermal cycling conditions were 10 min at 95°C followed by 40 repeats of 15 sec at 95°C and 1 min at 60°C. To monitor DNA contamination, control reactions without the RNA template were performed in triplicate and one reaction without the reverse transcriptase enzyme was carried out per tissue sample.

Relative quantification (RQ) was obtained using the 2^−ΔΔCt^ method, adjusting the AR and Cx43 mRNAs expression to the expression of GAPDH mRNA and taking the adjusted expression in the control group as reference (RQ = 1) [24]. Three independent experiments were performed, each in triplicate with tissues prepared from different animals. 

### 2.5. Western Blot Analysis

Prostate fragments of control and flutamide-treated boars were homogenized on ice with a cold RIPA buffer (Sigma-Aldrich), sonicated, and centrifuged at 10,000 ×g for 20 min at 4°C as described previously [8]. Additionally, homogenates of prostates from prepubertally castrated pigs (*n* = 3) were used as positive controls for PCNA and cleaved caspase 3 analyses. Aliquots were assayed for protein by the Bradford dye-binding procedure with BSA as a standard [25]. Thereafter, 25 *μ*g of protein was solubilized in a sample buffer (Bio-Rad Labs.) and heated at 99.9°C for 5 min. After denaturation, proteins were separated by sodium dodecyl sulfate polyacrylamide gel electrophoresis (SDS-PAGE) in 10% (vol/vol) resolving gels under reducing conditions. Separated proteins were transferred onto polyvinylidene difluoride membranes (Merck Millipore, Darmstadt, Germany) using a wet blotter in the Genie Transfer Buffer (pH 8.4) for 90 min at a constant current of 250 mA. Nonspecific binding sites were blocked with a solution of nonfat dry milk (5%, wt/vol) containing 0.1% Tween 20 (vol/vol), and the membrane was incubated in rabbit polyclonal antibody against AR (1 : 5000; Santa Cruz Biotechnology, Santa Cruz, CA, USA), rabbit polyclonal antibody against Cx43 (1 : 4000; Sigma-Aldrich), rabbit polyclonal antibody against caspase 3 (1 : 1000; Cell Signaling Technology, Beverly, MA, USA), and mouse monoclonal antibody against PCNA (1 : 500; Merck Millipore) for 2 h at room temperature. Thereafter, the membranes were washed briefly with TBST (0.05 M Tris-HCl, 0.15 M NaCl, 0.1% Tween 20, pH 7.6) and incubated in a goat anti-mouse or goat anti-rabbit IgG linked to the horseradish-peroxidase (1 : 3000; Vector Lab., Burlingame, CA, USA) for 1 h at room temperature. Immunoreactive proteins were detected by chemiluminescence with Western Blotting Luminol Reagent (Santa Cruz Biotechnology), and images were captured with a ChemiDoc XRS+ System (Bio-Rad Labs.). All immunoblots were stripped with stripping buffer containing 62.5 mM Tris-HCL, 100 mM 2-mercaptoethanol, and 2% SDS (wt/vol) (pH 6.7) at 50°C for 30 min and incubated in rabbit polyclonal antibody against *β*-actin (dilution, 1 : 3000; Sigma-Aldrich) which served as a loading control. Each data point was normalized against its corresponding actin data point. Molecular masses were estimated by reference to standard proteins (Fermentas, GmbH, St. Leon-Rot, Germany). To obtain quantitative results, immunoblots were analyzed using Image Lab 2.0 (Bio-Rad Labs.).

### 2.6. Morphological and Histomorphometrical Analysis

Hematoxylin and eosin staining of paraffin-embedded prostates was performed by a routine protocol. Morphological analyses were performed on Nikon Eclipse Ni microscope (Nikon Co., Tokyo, Japan), and the microscopic fields were digitized. 

The microscope with a ×10 ocular and a ×20 objective was used for the histomorphometrical measurements. Detailed analyses were performed on random images of 30 histological fields per experimental group with the use of NIS-Elements Microscope Imaging Software (Nikon Co.). Epithelium height, as well as the area of luminal, epithelial, and stromal compartment was measured, and relative proportions among the prostate components were calculated.

### 2.7. Immunohistochemistry and Immunofluorescence

 Prostate sections were cleared in xylene and rehydrated in a series of ethanol grade. To achieve antigen retrieval the slices were immersed in 10 mM citrate buffer (pH 6.0) and heated for 8 min in the microwave oven (750 W).

For AR and Cx43 immunostaining, endogenous peroxidase activity was neutralized with methanol containing 0.3% H_2_O_2_ for 10 min whereas nonspecific binding sites were blocked with 10% nonimmune goat or horse serum (vol/vol) for 30 min at room temperature. Then, prostate sections were incubated overnight at 4°C in a humidified chamber with appropriate primary antibody: rabbit polyclonal antibody against AR (1 : 250; Santa Cruz Biotechnology), or rabbit polyclonal antibody against Cx43 (1 : 50; Sigma-Aldrich). Next, biotinylated secondary antibody, goat anti-rabbit IgG (1 : 400; Vector Lab.), was applied for 60 min. After each step in these procedures, sections were carefully rinsed with Tris-buffered saline (TBS; 0.05 M Tris-HCl, 0.15 M NaCl, pH 7.6); the antibodies were also diluted in TBS buffer. The staining was developed using avidin-biotinylated horseradish peroxidase complex (ABC/HRP; 1 : 100; VECTASTAIN Elite ABC Reagent, Vector Lab.) for 30 min followed by 0.05% 3.3′-diaminobenzidine tetrachloride (DAB; Sigma-Aldrich) in TBS containing 0.01% H_2_O_2_ and 0.07% imidazole for 6 min. Thereafter, sections were washed, slightly counterstained with Mayer's hematoxylin, dehydrated, and mounted using DPX mounting media (Sigma-Aldrich). All slides were processed immunohistochemically at the same time with the same treatment so that staining intensity among different sections of the prostate could be compared. Negative controls included sections incubated with 10% nonimmune goat or horse serum instead of primary antibody. All immunohistochemical experiments were repeated three times. Immunohistochemistry for AR and Cx43 was also performed using NovoLink Polymer Detection System (Novocastra Labs., Newcastle upon Tyne, UK), according to the manufacturer's instructions. Afterwards, sections were examined with a Leica DMR microscope (Leica Microsystems, Wetzlar, Germany) using Nomarski interference contrast. Since there were no differences in the staining localization and intensities between both methods used, only the results obtained by the former technique (ABC method) were presented and discussed below.

 For PCNA immunofluorescence, sections were first incubated overnight at 4°C in a humidified chamber with mouse monoclonal antibody against PCNA, clone PC-10 (1 : 1000; Merck Millipore). After being rinsed in TBST, the sections were incubated with fluorescent Alexa Fluor 488 goat anti-mouse antibody (Invitrogen, Carlsbad, CA, USA) at 1 : 100 dilution for 1.5 h in the dark. Finally, the slides were mounted in Vectashield medium for fluorescence with 4′6-diamidino-2-phenylindole (DAPI; Vector Labs.) and viewed under a Zeiss confocal laser scanning microscope LSM510 (Carl Zeiss GmbH, Jena, Germany).

### 2.8. Qualitative and Quantitative Evaluation of the Immunohistochemical Reactions

Immunohistochemical staining for both antigens, Cx43 and AR, was evaluated qualitatively in at least 20 serial sections from each experimental group. The cells were considered immunopositive if brown reaction product was present in the cell nuclei, cytoplasm or appeared as signal between the cells. 

To evaluate the intensity of immunohistochemical reaction quantitatively, digital images of prostate sections were obtained using a Nikon DS-Fi2 Camera mounted on a Nikon Eclipse Ni microscope (Nikon Co.). Images were captured using a ×10 ocular and a ×20 objective. Image processing and analyses were performed using the public domain ImageJ software (National Institute of Health, Bethesda, MD, USA). The intensity of the immunohistochemical reaction was expressed as relative optical density (ROD) of diaminobenzidine brown reaction product and calculated using the formula described by Smolen [26]. A total number of 40 prostate sections (*n* = 10 per group) were subjected to image analysis and results of 10 separate measurements were expressed as mean ± SD.

### 2.9. Statistical Analysis

Each variable was tested by using the Shapiro-Wilk *W*-test for normality. Homogeneity of variance was assessed with Levene's test. Since the distribution of the variables was normal and the values were homogeneous in variance, all statistical analyses were performed using one-way analysis of variance (ANOVA) followed by Tukey's *post hoc* comparison test to determine which values differed significantly from controls. The analysis was made using Statistica 10 software (StatSoft Inc., Tulsa, OK, USA). Data were presented as mean ± SD. Data were considered statistically significant at *P* < 0.05.

## 3. Results

### 3.1. Histology of the Prostates of Control and Flutamide-Exposed Boars

**Figure 1:**
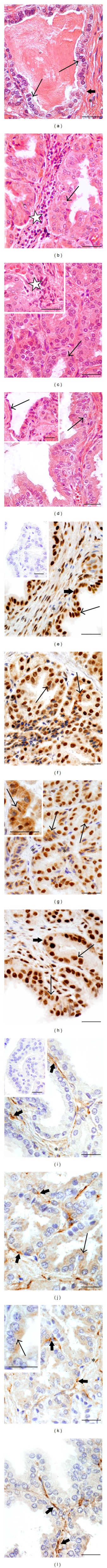
Morphology and immunohistochemical localization of AR and Cx43 in prostates of control and flutamide-exposed boars. Scale bars represent 20 *μ*m. ((a)–(d)) Morphology of the prostates. Prostatic tissue of control boars composed of fibromuscular stroma (asterisks) and acini lined with the epithelium containing basal (short arrows) and secretory cells (arrows) (a). Note, decreased size of luminal compartment ((b), (c)) and inflammatory foci in the stroma in GD20 and PD2 animals (white asterisks) ((b), insert in (c)). In PD90 group hyperplastic and dysplastic epithelial alterations (insert in (d)) are visible (d). ((e)–(h)) Immunohistochemical localization of AR. Typical distribution of AR to the nuclei of secretory (arrows), basal (short arrows), and stromal cells (asterisks) in the control group (e). Decreased staining intensity in the epithelium (arrows) and stroma (asterisks) of GD20, PD2 ((f), (g)) and to the lesser extent in PD90 boars ((h)). Note, immunonegative nuclei of stromal cells ((f), (g)) and cytoplasmic staining in some epithelial cells (insert in (g)) of GD20 and PD2 males. No signal was detected when anti-AR antibody was substituted by normal goat serum (insert in (e)). ((i)–(l)) Immunohistochemical localization of Cx43. The staining localized at the base of the epithelium and occasionally in the stroma of control prostates (i). Increased number and intensity of the immunopositive foci in flutamide-exposed pigs ((j), (k), (l)). Note the staining dispersed in the cytoplasm of secretory cells of GD20 and PD2 boars ((j), (k), insert in (k)). No signal was detected when anti-Cx43 antibody was substituted by normal goat serum (insert in (i)).

The prostate structure in control boars presented acini with simple cylindrical epithelium and fibromuscular stroma (asterisks) (Figure 1(a)). The secretory epithelium lining the prostatic acini was composed of a single layer of secretory cells (arrows) and a discontinuous layer of relatively few basal cells (short arrows). In GD20 and PD2 animals, reduction of acini size was observed (Figures 1(b) and 1(c)). Morphometric analysis revealed decrease of luminal compartment (*P* < 0.01), whereas epithelium height was unaltered when compared with the controls (Table 1). In the stroma, inflammatory foci were observed (white asterisks) (Figures 1(b) and 1(c)). In PD90 group dysplastic areas with variation in cell size and shape were occasionally observed (Figure 1(d), insert). In some areas, the epithelium had become thicker with agglomerated nuclei (arrows); however, no statistically significant differences in epithelial height were found when compared to control males (Table 1).

### 3.2. Localization of AR and Cx43 in the Prostates of Flutamide-Treated Boars

**Figure 2:**
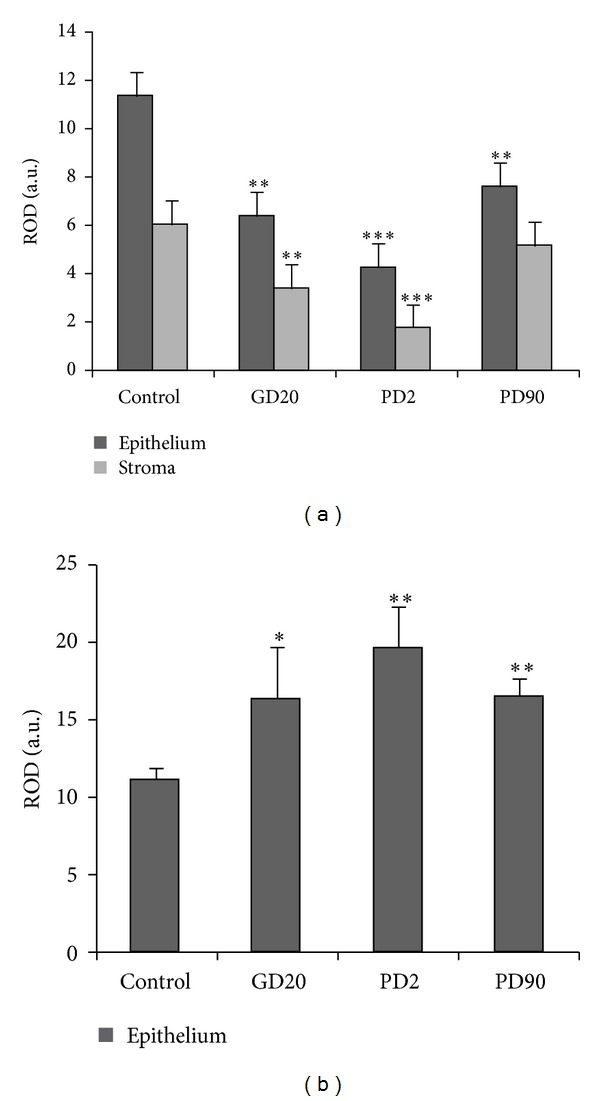
Quantitative analysis of immunohistochemical staining for AR and Cx43. Histograms of AR (a) and Cx43 (b) staining intensities expressed as relative optical density (ROD) of diaminobenzidine brown reaction products. Data expressed as mean ± SD. Significant differences from control values are denoted as *P < 0.05, **P < 0.01, and ***P < 0.001.

Immunostaining for AR was localized to both epithelial and stromal compartments of control and flutamide-exposed pigs (Figures 1(e)–1(h)). In the glandular epithelium of control pigs, the nuclei of secretory cells were strongly stained (arrows), whereas basal cells exhibited weaker signal (short arrows). In the stroma, immunostaining of variable intensity was detected (asterisks) (Figure 1(e)). The positive staining was also found in smooth muscle cells surrounding blood vessels (not shown). In the prostatic epithelium of GD20 and PD2 groups, the staining pattern presented heterogeneous but clearly less intense reaction (arrows) in comparison to the control group. In some epithelial cells a redistribution of AR from the nuclei to the cytoplasm was observed (Figure 1(f), insert in Figure 1(g)), whereas most stromal cells were negative (asterisks) (Figure 1). In PD90 boars the staining pattern was similar to that of control animals, but the intensity of the staining was slightly reduced (Figure 1(h)). Quantitative evaluation of the intensity of immunohistochemical staining, expressed as relative optical density (ROD), indicated the most pronounced decrease of AR immunostaining in PD2 pigs (*P* < 0.001) (Figure 2(a)).

In control boars immunohistochemical analysis revealed the presence of Cx43-positive cells predominantly at the base of the glandular epithelium, corresponding to the localization of basal epithelial cells (Figure 1(i)). Cx43 signal appeared as lines or clusters within the border between epithelial and stromal compartment (short arrows). Cx43-positive cells were also sparsely distributed within the stromal tissue (asterisks). Exposure of male pigs to flutamide clearly affected Cx43 immunoexpression in adult prostate. Number of Cx43-positive foci and intensity of the staining increased in these animals, as demonstrated by quantitative analysis (*P* < 0.05; *P* < 0.01) (Figure 2(b)). Moreover, in GD20 and PD2 groups cytoplasmic staining appeared in some epithelial cells of prostatic acini (arrows), which was not present in control boars (Figures 1(j) and 1(k)). In PD90 males distribution pattern of Cx43 was similar to that of control pigs, but immunopositive foci were more numerous (Figure 1(l)).

In the negative control tissue sections no immunopositive signal was found when the incubation was performed without the respective primary antibody (insert in Figures 1(e) and 1(i)).

### 3.3. Expression of mRNA and Protein for AR and Cx43 in the Prostates of Flutamide-Treated Boars

**Figure 3:**
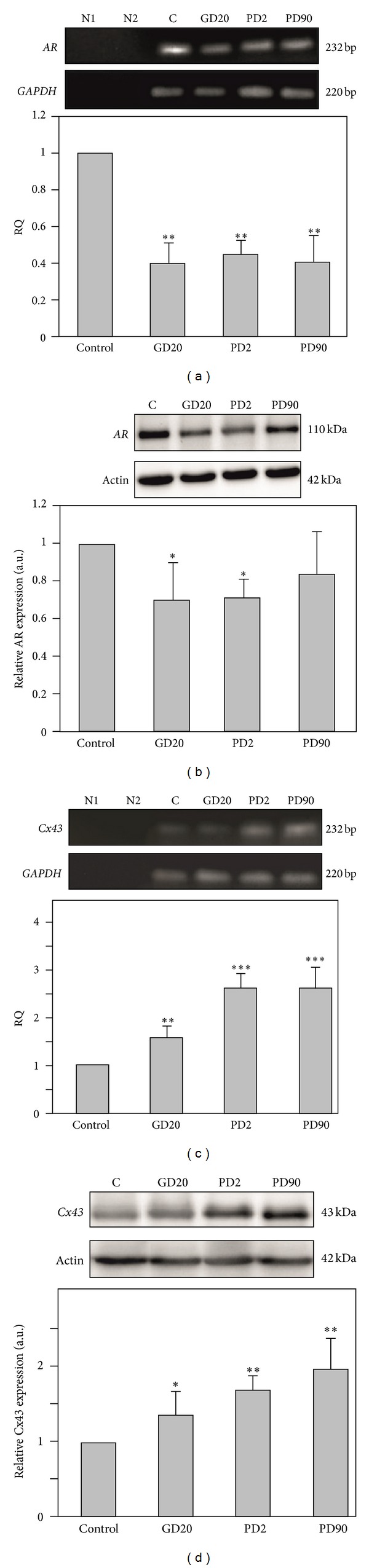
Androgen receptor and Cx43 mRNAs and protein expression in prostates of control and flutamide-exposed boars. ((a), (c)) Androgen receptor and Cx43 mRNAs expression. As an intrinsic control, the GAPDH mRNA level was measured in the samples. Representative gels electrophoresis of qualitative expression of AR (a), Cx43 (c) and GAPDH mRNAs. Line N1—negative control without cDNA template, line N2—negative control without reverse transcribed RNA. Relative expression of mRNA for AR (a) and Cx43 (c) determined using real-time RT-PCR analysis. Relative quantification (RQ) is expressed as means ± SD. Significant differences from control values are denoted as *P < 0.05, **P < 0.01, and ***P < 0.001. ((b), (d)) Androgen receptor and Cx43 protein expression. Representative immunoblots for AR (b) and Cx43 (d). Actin was used as a loading control, and each set of shown actin immunoblots corresponds to the target protein that was investigated within a given panel. The relative level of AR (b) and Cx43 (d) protein normalized against its corresponding *β*-actin. Data obtained from three separate analyses is expressed as mean ± SD. Significant differences from control values are denoted as *P < 0.05, **P < 0.01, and ***P < 0.001.

The expression of mRNA for AR and Cx43 in prostates of control and flutamide-exposed boars was assessed using the RT-PCR technique. Electrophoresis revealed PCR-amplified products of the predicted sizes: 242 bp for AR, 232 bp for Cx43, and 220 bp for GAPDH (Figures 3(a) and 3(c)). To determine the effect of flutamide on the expression of AR and Cx43 at the mRNA level, real-time RT-PCR analysis was performed (Figure 3). The analysis revealed statistically significant changes in AR mRNA levels in all flutamide-exposed groups, reaching about 2-fold decrease in mRNA expression compared with the control group (*P* < 0.01) (Figure 3(a)). In contrast, Cx43 mRNAs were upregulated following flutamide treatment, most markedly in PD2 and PD90 pigs (*P* < 0.001) (Figure 3(c)). No amplification was observed in all no-template and no-RT control analyses, indicating the specificity of the assays for mRNA.

Western blot analysis was performed to assess changes in the level of AR and Cx43 protein expression following flutamide exposure (Figures 3(b) and 3(d)). Immunodetectable AR and Cx43 proteins were observed as single bands near the 110 kDa and 43 kDa position, respectively, of the SDS gel in prostate homogenates of the control boars and those treated with flutamide. Densitometric analysis revealed significant decrease of AR expression in GD20 and PD2 groups (*P* < 0.05) (Figure 3(b)). In PD90 boars slight, not statistically significant reduction of AR protein level was found. Connexin 43 protein expression was increased in all flutamide-exposed groups; however, the most distinct effect was found in PD2 and PD90 males (*P* < 0.01) (Figure 3(d)).

### 3.4. Apoptosis and Proliferation in the Prostates of Flutamide-Treated Boars

To further investigate the effect of androgen signaling disruption, apoptosis, and proliferation of prostatic cells of control and flutamide-exposed males were determined using proliferating cell nuclear antigen (PCNA) as a proliferation marker and cleaved caspase 3 as an apoptotic marker. As the positive control a prostatic tissue from castrated pigs was included.

**Figure 4:**
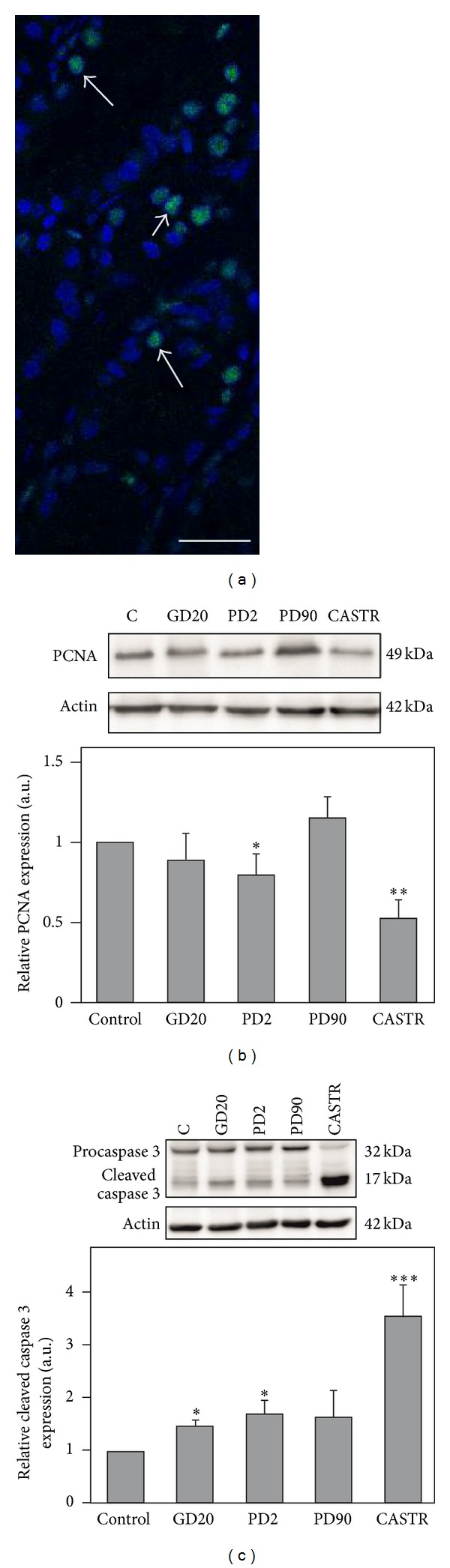
Proliferating nuclear antigen (PCNA) and caspase 3 protein expression in prostates of control and flutamide-exposed boars. (a) Immunofluorescent detection of PCNA in control prostate section (white arrows). Nuclei were counterstained with DAPI (blue). Scale bar represents 20 *μ*m ((b), (c)). Representative immunoblots for PCNA (b) and caspase 3 (c). The band at 32 kDa represents inactive proenzyme (procaspase 3) and the band at 17 kDa corresponds to active form (cleaved caspase 3). Actin was used as a loading control, and each set of shown actin immunoblots corresponds to the target protein that was investigated within a given panel. The relative level of PCNA (b) and cleaved caspase 3 (c) protein normalized against its corresponding *β*-actin. Data obtained from three separate analyses is expressed as mean ± SD. Significant differences from control values are denoted as *P < 0.05, **P < 0.01, and ***P < 0.001.

Proliferating cell nuclear antigen is an auxiliary protein of DNA polymerase delta involved in the control of eukaryotic DNA replication; therefore, its expression level reflects proliferative activity of a tissue [27]. Proliferating cell nuclear antigen expression was visualized by immunofluorescence (Figure 4(a)) and confirmed by Western blotting (Figure 4(b)). A single band at approximately 34 kDa corresponding to PCNA protein was detected in prostate homogenates of control and flutamide-treated pigs (Figure 4(b)). In GD20 and PD2 prostates reduction in PCNA protein expression was found; however, the effect was statistically significant only in PD2 group (*P* < 0.05). Treatment with flutamide during prepubertal period (PD90) results in a slight, not significant increase in PCNA level. In the prostates of adult castrated boars about 50% decrease of PCNA expression was observed (*P* < 0.01) (Figure 4(b)).

Proteolytic cleavage of procaspase 3 occurs to generate an active 17–19 kDa caspase 3 fragments (cleaved caspase 3), which target key modulators of the apoptotic pathway [28]. Using Western blot technique both forms of caspase 3 were detected in prostate homogenates of controls and flutamide-exposed boars (Figure 4(c)). The upper band at approximately 32 kDa corresponds to the procaspase 3, whereas lower band at 17 kDa corresponds to the cleaved caspase 3. Densitometric analysis revealed increase of cleaved caspase-3 level in the prostates of GD20 and PD2 pigs (*P* < 0.05), whereas in PD90 males no obvious changes were found when compared to the controls. In the prostates of castrated boars 3.5-fold increase of the caspase 3 cleavage was detected (*P* < 0.001) (Figure 4(c)).

## 4. Discussion

In the present study we used flutamide, pure androgen receptor antagonist, to block androgen action transiently during the period of sex differentiation (GD20) as well as during neonatal (PD2) and prepubertal development (PD90) of the boar. We found that disruption of androgen signaling during these periods may affect functioning of prostatic cells in adult animals by altering the AR and Cx43 genes expression as well as proliferation and apoptosis rates.

Histological analysis of prostates of flutamide-exposed males showed no significant changes in their gross appearance when compared to the prostates of vehicle-treated controls. However, the presence of inflammatory foci and reduction of acini size were observed in GD20 and PD2 males. Prostatic inflammation is often detected in adult prostate as a response after treatment with different antiandrogenic chemicals during early development, such as vinclozolin and phthalates [29, 30]. Although precise mechanisms involved in the induction of inflammatory changes following developmental exposures to endocrine disruptors are still not fully understood, multiple studies indicate that sex hormone imbalance may be the key factor in this process [31, 32]. Estrogens are considered as proinflammatory hormones, whereas testosterone exerts anti-inflammatory action on prostatic tissue [33]; thus, inflammatory foci in GD20 and PD2 boars are likely to result from decreased androgen: estrogen ratio described previously [34]. Altered action of sex hormones could also contribute to changes in prostatic acini morphology. Acini of GD20 and even more evident of PD2 males were usually smaller than these of control males, because of the reduction of the luminal compartment. Similar effect was reported in ventral prostates of rats exposed perinatally to di-n-butyl-phthalate (DBP), an environmental antiandrogenic chemical as well as in prostates of estrogenized castrated Mongolian gerbils [30, 35]. A common cause of reduced luminal area is altered secretory activity of epithelial cells. This activity is directly regulated by AR activation in the prostatic epithelium [36]. In the present study reduced expression of AR and its delocalization to the cytoplasm in some cells of prostatic epithelium of GD20 and PD2 boars was found. Cytoplasmic localization of AR may indicate receptor degradation and/or may be related to the reduced bioavailability of testosterone [37, 38]. Taken together, dysfunction of epithelial AR in flutamide-exposed pigs may be one of the trigger mechanisms contributing to the reduction of prostatic acinar lumen and size. 

Interestingly, in stromal cells of GD20 and PD2 males we found almost total loss of AR immunostaining. In fetal and perinatal development mesenchymal stromal cells are the main target for androgen action in the prostate, since epithelial cells are AR-negative until early postnatal period [39]. Therefore, it can be hypothesized that prenatal and neonatal exposure to flutamide originally affects stromal cell functions, altering their ability to respond to androgen signaling in adult male. This in turn may be the reason of disturbed prostatic epithelium functions in adulthood. Importance of androgen action in the stroma on normal structure and function of glandular epithelium was demonstrated by experiments performed on urogenital tissue recombinants from wild-type and androgen-insensitive mice [40]. The authors showed that many androgenic effects on prostatic epithelial development do not require epithelial AR and these effects are elicited by the paracrine action of AR-positive mesenchymal stroma. These observations were supported by recent studies on tissue-selective knockout mice with the AR gene deleted in stromal smooth muscle cells or stromal fibroblasts [41, 42]. Interestingly, in both knockout models reduced epithelial cell proliferation was described. Therefore, it is likely that decreased proliferation, as reflected by reduced expression of PCNA, found in prostates of PD2 boars and, in lesser extent, in GD20 boars is directly associated with altered expression of AR in the stromal tissue. Indeed, Perry and Tindall [43] showed that in human prostate cell line the antiandrogen, casodex, inhibited the mibolerone-stimulated increase in PCNA expression, suggesting that the androgenic induction of PCNA is mediated through the AR.

Caspase 3 activation is considered as an indicator of apoptosis induction, because different upstream pathways leading to apoptosis depend on caspase 3 cleavage [44]. Thus, in the present study apoptosis was assessed by evaluating the level of cleaved (active) form of caspase 3 in prostate homogenates. Since it is known that castration induces apoptosis of prostatic epithelial cells, to validate our results we included an analysis of cleaved caspase 3 expression in prostates of castrated pigs that served as a positive control. Indeed, the level of cleaved caspase 3 in prostates of castrated boars appeared to be 3.5-fold higher when compared to the vehicle-treated control males. In the prostates of GD20 and PD2 boars much less marked, but still statistically significant increase in the apoptosis activation was detected. This is in line with earlier studies showing that the fetal hormonal disruption induced by antiandrogens, such as flutamide, vinclozolin, and phthalates, gives rise to a long-term apoptosis in reproductive organs (testis, epididymis) in the adult male [45, 46]. Increased apoptosis was detected also in the testes and epididymides of adult boars treated with flutamide during prenatal and neonatal development [9, 10]. Therefore, our data support the concept of fetal and perinatal programming of adult cell apoptosis in reproductive tissues. Another possible explanation is that decreased testosterone level and reduced expression of AR in prostates of adult GD20 and PD2 males directly induce caspase cleavage, activating cell death pathways.

In PD90 boars the effects of flutamide treatment on prostate morphology and AR expression were less pronounced than those observed in GD20 or PD2 males. Prostatic acini were well developed with the lumen comparable to that of control pigs and there was no evidence of inflammatory changes. However, glandular epithelium was more folded with occasionally observed hyperplastic or dysplastic changes. Although AR mRNA level was clearly downregulated, the reduction of AR protein expression in PD90 males was less evident particularly in the stroma. The inconsistency observed between PCR and Western blot analysis of AR may suggest that stability of AR mRNA or rate of metabolic degradation of AR protein in the prostate of these animals was altered [47, 48].

Moreover, in PD90 boars no marked changes in PCNA and cleaved caspase 3 expression were detected. This is in line with the results by Gómez et al. [49], who showed that chemical castration induced by cyproterone acetate treatment during prepubertal period did not affect cell proliferation and apoptosis. Therefore, it is likely that effects of androgenic blockade during prepubertal period on cell proliferation and apoptosis are transient and are not maintained in adult boar prostate. However, it cannot be excluded that androgen action during prepubertal period is not necessary to control cell proliferation and to protect prostatic cells from apoptosis.

According to our knowledge Cx43 expression and distribution in the boar prostate have not been described yet. We used real-time RT-PCR and Western blot methods to reveal the presence of Cx43 mRNA and protein in prostate homogenates of adult intact boars. Immunohistochemical analysis demonstrated that in boar prostate Cx43 was localized predominantly in the basal region of glandular epithelium, similarly as it was observed in rat [14]. In contrary to punctuate signal dispersed within the secretory cell layer in guinea pig and stallion, no staining was detected in the secretory cells of prostatic acini of the boar, suggesting species-dependent distribution of Cx43 in prostatic epithelium [50, 51]. It is also possible that in porcine prostatic epithelium the amount of Cx43 in secretory cells is below the detection sensitivity of immunohistochemical methods used herein. 

Following prenatal and postnatal exposure to flutamide, Cx43 mRNA and protein levels markedly increased, predominantly in PD2 and PD90 males. Increased intensity of Cx43 staining and increased number of Cx43-positive foci were also evident in immunohistochemical sections. Previous studies on the rat model showed that Cx43 expression is directly regulated by testosterone in adult males [52]. The authors demonstrated that androgen deprivation induced by castration upregulated Cx43 mRNA and protein expression in the prostate, while androgen replacement resulted in restoration of Cx43 level to the values characteristic for normal (sham-castrated) rats. It is likely therefore that alterations of Cx43 expression found in our study are related to androgen signaling disruption due to AR downregulation and/or decreased testosterone concentration in adult boars following developmental exposure to flutamide. Increased testosterone aromatization to estradiol in flutamide-treated boars [34] could also contribute to enhanced Cx43 expression in prostatic cells as shown by Carruba et al. [53]. 

Interestingly, in GD20 and PD2 males diffused staining appeared in the cytoplasm of secretory epithelial cells, whereas in the controls they were immunonegative, indicating altered phenotype of secretory cells in flutamide-exposed groups. Habermann et al. [14] demonstrated that differentiation of prostatic epithelium during postnatal development is associated with progressive loss of Cx43 expression. Thus, the presence of Cx43 in secretory epithelial cells of boars treated with flutamide during fetal and neonatal period may reflect altered differentiation of prostatic epithelium. 

However, it cannot be excluded that local inflammatory changes found in the prostates of GD20 and PD2 boars contribute to the alterations in Cx43 expression. The relationship between Cx43 expression and inflammation was demonstrated to date in several organs, including liver, kidney, testis, epididymis, and brain [10, 54–57]. Furthermore, it was established that proinflammatory cytokines, such as tumor necrosis factor and interleukin-1, modulate the cell-to-cell communication and connexin 43 levels *in vitro* [58].

It is worth noting that Cx43 and AR alterations in the prostates demonstrated in the present study are different than alterations recently observed in testes and epididymides of flutamide-exposed boars [9, 10]. It is likely, therefore, that Cx43 and AR genes expression during development is differentially regulated by androgens in different tissues of porcine male reproductive system. 

The question arises as to the causal link between the transient developmental exposure to flutamide and the adult prostate disorders. Several studies indicate that upstream mechanisms leading to the long-term alterations observed in adult prostate may be related to the epigenetic changes, such as DNA methylation and histone modifications. It was reported that changes in methylation states of several genes induced by exposure to antiandrogen vinclozolin during the period of sex determination are correlated with disease state in numerous organs of adult rats, including prostate [5, 59]. The role of epigenetic factors was also demonstrated by Ho et al. [60], who found altered methylation pattern of phosphodiesterase type 4 in adult rat prostate following neonatal treatment with estradiol. For Cx43, epigenetic effect of different toxicants in the liver has also been documented [61]. In prostate cancer cells, Hernandez et al. [62] found that Cx43 was induced as a result of hyperacetylation of histones H4 surrounding the AP-1- and Sp1-responsive gene elements. It was also established that CpG methylation and histone acetylation may play important roles in the regulation of the AR, especially in prostate cancer cells [63, 64]. Importantly, recent studies indicate that prostate stem/progenitor cells may undergo life-long reprogramming, as a consequence of developmental exposures to endocrine disrupting chemicals [65].

Finally, there are two limitations that need to be taken into account when considering the study and its contributions. The first is relatively small number of experimental animals used in the study. Owing to the significant physiological, biochemical, anatomical, and genomic similarities between pigs and humans, the pig provides a uniquely relevant biomedical model for human, having a distinct advantage over rodents [66]. The expense of experiments, performed on pigs, did not allow to conduct studies on large group of animals. However, in the present study the responses of the individual animals to experimental conditions within each experimental group were very homogeneous. Furthermore, to validate the results each analysis was performed in triplicate using several complementary techniques.

Second limitation is the assessment of the effects of flutamide exposure on the prostate only at adulthood. It should be stressed, however, that in the previous studies from this laboratory no changes in the testes of neonatal pigs after fetal exposure to flutamide were found, whereas in prepubertal males seminiferous tubules were affected occasionally [18, 19]. The most severe alterations were clearly found in adult testes [9]. Those observations suggest that most effects of transient developmental exposure to flutamide on pig testis are not revealed until adulthood. Thus, we anticipate that such long-term effect may also occur in the prostate. To test directly short-term effects of flutamide action, *in vitro* studies have recently been undertaken.

Altogether the results presented herein indicate that in boar androgen action during early fetal and neonatal periods plays a fundamental role in the maintenance of normal phenotype and functions of prostatic cells at adulthood. Downstream effects of the blockade of AR activation in these critical periods include changes in the expression and cellular localization of AR and Cx43, as well as alterations of proliferation and apoptosis ratio. Moreover, we demonstrated that modulation of Cx43 expression in the prostate could serve as a sensitive marker of hormonal disruption on different developmental stages, since deregulation of this protein was found in all experimental groups.

## Figures and Tables

**Table 1:** Morphometric analyses of prostate sections from control and flutamide-treated boars (*n* = 30 fields/group).

	Control	GD20	PD2	PD90
Epithelial compartment (%)	73.44 ± 4.43	79.45 ± 7.91	82.77 ± 5.36	63.13 ± 4.34
Luminal compartment (%)	17.17 ± 3.11	5.07 ± 0.34**	5.41 ± 1.35**	22.25 ± 4.42
Stromal compartment (%)	9.39 ± 1.46	15.27 ± 7.90	11.61 ± 4.34	14.62 ± 1.48
Epithelium height (*µ*m)	15.35 ± 2.80	13.12 ± 2.14	14.25 ± 2.22	14.51 ± 4.15

Data are expressed as means ± SD. Significant differences from control values are denoted as **P < 0.01.
